# EMERGING COHORT DISPARITIES IN MULTIMORBIDITY IN US ADULTS ENTERING EARLY OLDER ADULTHOOD

**DOI:** 10.1093/geroni/igad104.0760

**Published:** 2023-12-21

**Authors:** Nicholas Bishop, Steven Haas, Ana Quiñones

**Affiliations:** University of Arizona, Tucson, Arizona, United States; Penn State, University Park, Pennsylvania, United States; Oregon Health and Science University, Portland, Oregon, United States

## Abstract

Recent evidence of generational disparities in multimorbidity risk among older adults indicates earlier transitions into multimorbidity and greater disease burden for those born to later cohorts. As costs of managing chronic disease will rise with population aging, it is imperative to identify whether cohort effects in multimorbidity extend to cohorts just entering early older adulthood. We use representative data from the Panel Study of Income Dynamics to model cohort trends in multimorbidity from 1999–2019 in adults who were 50 years of age or older at any observational period (n = 4,975; 40,822 person-observations). Multimorbidity was assessed as a count of nine chronic conditions (stroke, hypertension, diabetes, cancer, lung disease, heart problems, emotional problems, arthritis, mental loss). Weighted Poisson mixed models were used to estimate multimorbidity trajectories adjusted for fixed effects of cohort, linear and quadratic age, and sociodemographic covariates. The model accounted for repeated measures though inclusion of a random effect for within-person error covariance across wave. After adjustment, those born to the Early and Late Baby Boom cohorts (b. 1946–1955 and b. 1956–1964, respectively), as well as those born to the early Gen X cohort (b. 1965–1972), reported significantly greater multimorbidity burden than those born to the late depression-era (b. 1931–1942). These findings extend evidence of cohort effects in multimorbidity in U.S. older adults, indicate these problematic trends apply to U.S. adults entering early older adulthood, and suggest practitioners and policymakers should prepare for an aging population with expanding disease burden.

